# Lithium in borderline personality disorder: neuroplasticity, anti-inflammatory action, and therapeutic potential

**DOI:** 10.3389/fnbeh.2026.1707007

**Published:** 2026-03-19

**Authors:** Leah Simon, Halford Warlick, Armena Jafarmadar, Ernesto Joubran, Marianne Koleng, Christina Regine Owens-Charles, Priyanka Ramanathan, Jorge Rubinos Rodriguez, Stephanie Singer, Vincent S. Gallicchio

**Affiliations:** 1 Dr. Kiran C. Patel College of Osteopathic Medicine, Nova Southeastern University, Davie, FL, United States; 2 The Chicago School, Chicago, IL, United States; 3 Department of Biological Sciences, College of Science, Clemson University, Clemson, SC, United States

**Keywords:** anxiety, borderline personality disorder, lithium, neurobiology, therapy

## Abstract

The use of lithium (Li) for borderline personality disorder (BPD) is limited as the result of a lack of robust clinical evidence with few clinical studies reported. Although a few studies provide supportive evidence for Li use in addressing certain symptoms such as anger management, impulsivity, and inducing self-harm. Therapies for BPD are psychosocial in nature and serve as the primary treatment for BPD. Based on prior clinical management of lithium use in mental health disorders, providers should investigate its usage in the treatment of BPD. The topic is the subject of this review.

## Introduction

Borderline personality disorder (BPD) is a severe and complex psychiatric condition characterized by pervasive affective instability, impulsivity, disturbed interpersonal relationships, and elevated risk of self-harm and suicide. Prevalence of the disorder is estimated at 0.5–5.9% in the general population, with up to 10% of patients completing suicide, representing a nearly 50-fold increased risk compared to the general population (Leichsenring et al., 2011).

Psychotherapy, particularly dialectical behavior therapy (DBT), remains the cornerstone of treatment for BPD (NICE, 2009; APA, 2023). Pharmacological interventions are considered adjunctive and are typically prescribed to target symptom clusters such as affective lability, impulsivity, or transient psychotic-like states (Lieb et al., 2010).

Pharmacotherapy in BPD continues to be controversial, and no medications have been approved by regulatory agencies for this indication (Gartlehner et al., 2021). Controversial in part because there are no FDA-approved medications for BPD itself, leading to a reliance on a symptom-based approach that has a concurrent inconsistency of effectiveness. Other reasons for this controversial approach are the high incidence of misdiagnosis, leading to incorrect treatment, and the risk of co-occurring conditions that complicate the selection of alternative drug choices, coupled with safety concerns due to BPD patients’ high-risk for suicide.

Lithium, long established as a mood stabilizer in bipolar disorder (Albano and Gallicchio, 2023; Gupta and Gallicchio, 2023) has been investigated in BPD for its potential to reduce impulsive aggression, suicidality, and mood instability (Cipriani et al., 2013; Berghöfer, 2013; Tondo and Baldessarini, 2024). These properties make it an intriguing candidate for addressing the complex symptomatology of BPD. This review examines lithium’s pharmacological mechanisms, evaluates the available clinical evidence for use in BPD, and considers its role within treatment guidelines with the aim of clarifying lithium’s potential relevance for BPD and identifying priorities for future research.

The evidence for lithium in BPD is often extrapolated from its use in bipolar disorder, a separate diagnosis that shares many of the symptoms with BPD. Lithium can be toxic clinically especially when administered in high does; however, this concern is now mitigated based on the development of low dose formulations and much improved clinical monitoring such as bedside/out-patient methodology. Lithium is not a standard, widely recommended treatment for BPD due to limited evidence and potential risks. It may be considered in specific cases where other treatments have failed, particularly for symptoms of impulsivity, anger, or aggression. The decision to use lithium should be made on an individual basis by a qualified health care professional, and it should always be used as an adjunct to psychotherapy.

### Neurobiology of borderline personality disorder

Borderline personality disorder (BPD) is increasingly understood as a disorder of corticolimbic circuitry, involving abnormalities in brain regions that regulate affect, impulse control, and stress reactivity. The most consistent neuroimaging findings indicate hyperactivation of the amygdala and hippocampal regions during emotional processing tasks, as well as impaired amygdala habituation to repeated negative stimuli (Leichsenring et al., 2024). These alterations are thought to underlie the affective instability and heightened emotional sensitivity observed clinically.

Structural alterations have been reported, though results vary across studies. Earlier meta- analyses identified reductions in hippocampal and amygdala volumes, findings often attributed to early life stress and trauma (Nunes et al., 2009). More recent and comprehensive analyses, however, did not confirm consistent gray matter changes, highlighting heterogeneity in patient samples and imaging methods (Leichsenring et al., 2024). This suggests that structural abnormalities may be present in subsets of patients but are not universally observed across all individuals with BPD.

Prefrontal cortical involvement has yielded inconsistent results across studies. While some structural MRI investigations describe reduced gray matter density or hypoactivation in dorsolateral and orbitofrontal regions, large-scale reviews have not confirmed consistent abnormalities (Leichsenring et al., 2024). A recent task-based fMRI meta-analysis found that BPD patients exhibited hyperactivation of medial prefrontal and anterior cingulate regions during social cognition tasks, while broader task analyses additionally identified amygdala hyperreactivity and inferior frontal alterations (Schurz et al., 2024). These findings suggest that prefrontal alterations in BPD are heterogeneous and may be task-dependent rather than a consistent deficit across studies.

Dysregulation of the hypothalamic–pituitary–adrenal (HPA) axis is another frequently replicated finding. Meta-analytic data indicate a blunted cortisol response to psychosocial stress in BPD, with evidence of altered basal cortisol secretion. Altered HPA axis activity in BPD is often interpreted within an allostatic load model, in which chronic stress exposure leads to long-term dysregulation of stress responses and diminished physiological flexibility. These stress-related abnormalities overlap with findings in other stress-related disorders, but meta-analyses suggest that they are especially pronounced in BPD patients with a history of early trauma (Drews et al., 2019; Leichsenring et al., 2024). The convergence of limbic hyperreactivity, variable prefrontal regulation, and stress-system abnormalities underscores the need for interventions that can address both neural circuit function and stress-related biology in BPD. This framework provides a basis for considering pharmacologic strategies with established effects on neuroplasticity and stress modulation.

### Lithium: pharmacology and clinical profile

Lithium was first introduced to psychiatry following Cade’s seminal 1949 report describing its antimanic effects (Cade, 1949). It is now widely recognized as one of the most effective mood stabilizers for bipolar disorder, with additional anti-suicidal benefits (Cipriani et al., 2013). Beyond mood stabilization, lithium has widespread neurobiological effects that are relevant to the pathophysiology of BPD.

### Mechanisms of action

Lithium exerts pleiotropic effects at the molecular and cellular levels. Among its primary mechanisms is inhibition of glycogen synthase kinase-3β (GSK-3β) which activates the Wnt/β-catenin pathway and promotes cell proliferation, synaptic plasticity, and neurogenesis (Quiroz et al., 2004; Rowe and Chuang, 2004; Young, 2009). It also modulates inositol-phosphate signaling and increases expression of neurotrophic factors including brain-derived neurotrophic factor (BDNF) and vascular endothelial growth factor (VEGF), thereby supporting neuronal survival and dendritic remodeling (Chiu and Chuang, 2013; Malhi et al., 2013; Young, 2009). In addition, lithium upregulates the anti-apoptotic protein Bcl-2, which enhances mitochondrial function and neuronal survival, with recent evidence in human neuronal cells (Paul et al., 2020; Young, 2009). These mechanisms are thought to underpin both its neuroprotective capacity and its unique ability to reduce suicide risk (Smith and Cipriani, 2017).

Preclinical studies confirm these effects. Fiorentini et al. (2010) demonstrated in a transgenic Alzheimer’s model that chronic lithium treatment enhanced hippocampal progenitor proliferation, improved survival of newborn neurons, reduced neuropathology, and restored cognitive function. These findings provide mechanistic support for lithium’s ability to increase hippocampal volume and counteract disease-related atrophy. Complementary human imaging studies extend this evidence: long-term lithium exposure is associated with bilateral hippocampal volume increases in bipolar disorder patients (Yücel et al., 2007), and meta-analytic data suggest that lithium normalizes hippocampal size compared to unmedicated patients (Hajek et al., 2012). Taken together, these findings support lithium’s capacity to reverse or mitigate hippocampal volume loss, a compelling mechanism given the evidence for hippocampal deficits in BPD.

### Anti-inflammatory and gene-regulatory effects

In addition to its neurotrophic properties, lithium exerts broad immunomodulatory and anti- inflammatory actions. It reduces the expression of proinflammatory cytokines such as TNF-α and IL-6, suppresses microglial activation, and modulates immune signaling pathways (Chiu and Chuang, 2013; Rowe and Chuang, 2004; Young, 2009). Lithium also protects against oxidative stress by enhancing endogenous antioxidant defenses, including glutathione and superoxide dismutase activity, thereby limiting free radical-mediated neuronal injury (Quiroz et al., 2004; Chiu and Chuang, 2013). While oxidative imbalance has been consistently linked to mood and neurodegenerative disorders, its role in BPD is less clearly defined. What has been implicated is oxidative imbalance contributes to neural damage, mood regulation disruption, and impulsivity linked via increase inflammatory markers TNF-α and interleukin-6 (IL-6). Nonetheless, lithium’s ability to regulate overlapping stress and inflammation-related pathways may contribute to its potential therapeutic relevance in BPD. At the genomic level, lithium influences epigenetic regulation, such as histone acetylation and DNA methylation, which shift transcriptional programs toward neuroprotection, stress resilience, and suppression of inflammatory cascades (Chiu and Chuang, 2013; Malhi et al., 2013).

### Pharmacokinetics

Lithium is rapidly absorbed after oral administration, with immediate-release formulations reaching peak plasma concentrations within 0.25–3 h, while sustained-release formulations peak between 2 and 6 h. It has a distribution volume of approximately 0.7–1 L/kg and exhibits minimal binding to plasma proteins. Unlike many psychotropic agents, lithium does not undergo hepatic metabolism. Instead, lithium is excreted almost entirely in unchanged form via the kidneys. Around 80% of filtered lithium is reabsorbed in the proximal tubule, a process that parallels sodium handling, making serum lithium levels sensitive to hydration status and sodium balance. The elimination half-life ranges between 18 and 36 h, extending further in elderly individuals or those with renal impairment (Chokhawala et al., 2025).

### Therapeutic considerations

Lithium has a narrow therapeutic index. Serum concentrations of 0.8–1.2 mmol/L are typically required for mood stabilization, while lower ranges (0.1–0.4 mmol/L) may suffice for neuroprotection or suicide prevention (Malhi et al., 2017). Chronic administration is associated with adverse effects, including renal impairment, hypothyroidism, tremor, weight gain, and cognitive slowing, necessitating ongoing monitoring of serum lithium, renal function, and thyroid parameters (McKnight et al., 2012).

### Lithium in borderline personality disorder

#### Evidence from clinical trials

The direct evidence base for lithium in BPD is limited. The only randomized controlled investigation to date is a double-blind, placebo-controlled crossover trial involving 10 patients. Although the study found no statistically significant benefit compared to placebo on depressive symptom scales, clinicians noted improvements in anger and suicidality in most participants during lithium treatment (Links et al., 1990). These findings not only highlight lithium’s potential relevance for addressing the core affective and behavioral dysregulation of BPD but also underscore the limitations of early work due to sample size, short duration, and reliance on secondary clinician-rated impressions.

#### Narrative reviews and meta-analyses

Despite the paucity of direct clinical trials, narrative reviews have consistently reported that lithium reduces impulsivity, aggression, and self-harm behaviors in BPD. These effects are likely mediated through serotonergic, dopaminergic, and opioid modulation (Leichsenring et al., 2011). Systematic reviews and meta-analyses of mood stabilizers in BPD suggest modest benefit for irritability and self-injurious behavior, but emphasize that larger, high-quality randomized controlled trials are lacking (Lieb et al., 2010; Stoffers et al., 2010). More recent evidence syntheses reaffirm this conclusion. Gartlehner et al. (2021) found no robust support for any pharmacological treatment in BPD, though certain subgroups, particularly those with prominent affective instability or impulsive aggression, may derive symptomatic relief from mood stabilizers, including lithium. Gartlehner et al. (2021) concluded despite the common use of pharmacotherapies for patients with BPD, only low-quality evidence is available to guide clinicians. Use of lithium was not discussed in their review, thus leaving open the possibility for its testing. Overall, the efficacy of pharmacotherapies to improve BPD is limited to improvement of individual symptoms but not the condition overall. Even for the improvement of symptoms, the certainty of evidence is low. Future research needs to conduct unbiased, adequately powered trials that take potential differences in subgroups into consideration and focus on patient-relevant health outcomes, such as social functioning or clinically important improvements of symptoms that matter most to patients with BPD.

#### Clinical guidelines

Clinical guidelines consistently recommend psychotherapy as the first-line treatment for BPD, with pharmacotherapy, potentially should include investigating lithium, considered only adjunctive. The National Institute for Health and Care Excellence (NICE, 2009) and the American Psychiatric Association (APA, 2023) advise limiting psychotropic medications to short-term, symptom-targeted use, particularly in the context of comorbid mood or anxiety disorders. Neither guideline specifically endorses lithium, but both acknowledge that mood stabilizers may be considered when impulsivity, aggression, or affective lability predominate; however, further clinical evaluation is warranted.

#### Neurobiological and structural rationale

Beyond symptomatic effects, lithium’s relevance in BPD is supported by structural and mechanistic parallels with bipolar disorder. Neuroimaging meta-analyses demonstrate hippocampal and amygdala volume reductions in BPD, abnormalities also observed in bipolar disorder (Nunes et al., 2009). In bipolar patients, long-term lithium treatment is associated with bilateral hippocampal volume increases (Yücel et al., 2007) and normalization of hippocampal size relative to unmedicated patients (Hajek et al., 2012). By inhibiting glycogen synthase kinase-3β (GSK-3β), upregulating brain-derived neurotrophic factor (BDNF), and enhancing expression of the neuroprotective protein Bcl-2, lithium promotes hippocampal neurogenesis, dendritic arborization, and neuronal survival (Malhi et al., 2013). These actions could counteract the stress-related hippocampal atrophy and dysregulation of corticolimbic circuits that are implicated in BPD pathophysiology.

### Lithium versus non-lithium pharmacological interventions

As mentioned, pharmacotherapy primarily serves an adjunctive role to psychotherapy in the treatment of BPD. Lithium has been used for aggressive or impulsive behaviors and suicidality, although evidence supporting its use in BPD is limited (Almeida and Sanches, 2024). Non- lithium pharmacological interventions, most notably antidepressants and anticonvulsants, are also prescribed in a symptom-targeted and adjunctive capacity. Like lithium, these treatments provide supportive management of BPD-related symptoms and comorbidities (Gartlehner et al., 2021).

#### Antidepressants

Antidepressants such as selective serotonin reuptake inhibitors (SSRIs) are widely prescribed for individuals with BPD despite discrepancies among clinical guidelines (Pascual et al., 2023).

These medications are typically prescribed to address BPD-related symptoms of affective dysregulation and impulsivity (Gartlehner et al., 2021). However, a 12-week double-blind, placebo-controlled study involving 20 patients with BPD found no significant post-treatment differences in patients who received fluoxetine, an SSRI, in addition to dialectical behavior therapy (Simpson et al., 2004). Overall, the evidence supporting antidepressant use in BPD is limited, yet antidepressants remain the most frequently prescribed pharmacological treatment among psychiatrists (Pascual et al., 2021).

#### Anticonvulsants

Anticonvulsants including topiramate and lamotrigine have also been studied for targeting BPD-.

associated effects of affective instability and impulsive behavior (Gartlehner et al., 2021). Smaller randomized controlled trials have shown favorable results in reduction of anger and aggression in BPD patients. For example, a randomized, double-blind, placebo-controlled study with 14 female subjects diagnosed with BPD found lamotrigine to be appropriate and effective for reducing aggressive characteristics after 8 weeks of treatment (Tritt et al., 2005). An 18-month follow-up of this study confirmed that these improvements were sustained (Leiberich et al., 2008). Similarly, an 8-week study of 42 males with BPD found improved anger scales on the State–Trait Anger Expression Inventory (STAXI) with topiramate (Nickel et al., 2005), and these effects persisted at 18 months with good tolerability (Nickel and Loew, 2008).

Current evidence is insufficient to establish antidepressants or anticonvulsants as effective treatments for BPD. No specific pharmacotherapy has been approved as first-line treatment to date. Rather, these medications are recommended and often prescribed off label to target specific symptoms in BPD patients (Gartlehner et al., 2021; Pascual et al., 2021).

Lithium is not a first-line treatment for BPD and to date it has been demonstrated to have a limited role, with evidence primarily suggesting potential benefits for reducing impulsivity, aggression, and suicide risk rather than overall symptom management (Pascual et al., 2023). When used, it is typically in conjunction with psychotherapy or for BPD with comorbid bipolar spectrum disorders. Determining the appropriate lithium dosage for managing symptoms associated with BPD requires careful medical evaluation and monitoring. Dosage is highly individualized and depends on various factors, including the specific symptoms being addressed, the individual’s overall health, and the presence of any comorbid conditions.

Lithium is sometimes considered for certain BPD-related symptoms, such as affective instability, impulsivity, aggression, and suicidal behaviors, particularly when these symptoms are prominent (Almeida and Sanches, 2024). However, its effectiveness for core BPD features can be limited. When lithium is used, the goal is often to find a dosage that effectively manages the target symptoms while minimizing potential side effects. This typically involves regular blood tests to monitor lithium levels and ensure they remain within a safe range. The therapeutic range for lithium can vary depending on the condition being treated. It is crucial to understand that lithium therapy requires close medical supervision due to its narrow therapeutic index and potential for serious side effects, including weight gain, tremors, nausea, diarrhea, and impacts on kidney and thyroid function.

### Lithium, neurogenesis and BPD

Lithium’s role in BPD involves promoting neural plasticity and neurogenesis, particularly in the hippocampus, counteracting stress effects, and stabilizing mood by influencing growth factors (like BDNF, NGF), gene expression, and key molecular pathways (like Wnt/β-catenin, GSK-3β), potentially increasing hippocampal volume and improving function, though it’s not FDA-approved for BPD and psychotherapies remain primary treatments. How lithium promotes neurogenesis and plasticity has been elucidated based on the following observations:

- Increases neurogenesis: Studies show lithium can boost the production of new neurons (neuroblasts) and glial cells in the human hippocampus (Yücel et al., 2007).
- Enhances neurotrophic factors: It increases levels of brain-derived neurotrophic factor (BDNF) and nerve growth factor (NGF), which are crucial for neuron survival and growth (Malhi et al., 2013).
- Blocks stress effects: Lithium helps prevent stress-induced damage and volume loss in brain areas like the hippocampus and amygdala, facilitating neural repair (Hajek et al., 2012).
- Activates protective pathways: It promotes pathways like Wnt/β-catenin, which are vital for neural plasticity, and inhibits GSK-3β, a key enzyme involved in aging and cell signaling (Malhi et al., 2013).
- Modulates gene expression: Lithium can turn on genes for neuroprotection and plasticity while suppressing those linked to inflammation and neurodegeneration.

Impact on BPD and brain structure

- Increased hippocampal volume: Chronic lithium use is linked to larger hippocampal volumes in BPD patients, a region often affected by stress.
- Restores connectivity: It can increase connectivity, for example, between the amygdala and medial prefrontal cortex, correlating with mood stabilization.

Clinical relevance for BPD

- While lithium is primarily used for Bipolar Disorder, its neuroplastic and neurogenic effects are being studied for BPD, though psychotherapies remain the cornerstone of BPD treatment, notes a 2013 review in National Institutes of Health.
- Researchers are investigating how lithium’s effects on genes and brain structure contribute to its therapeutic potential in mood disorders.

## Conclusion

In summary, although only a limited number of clinical studies have investigated lithium use in BPD, available evidence suggests that further evaluation is warranted. The few studies conducted so far have involved relatively small sample sizes, making it difficult to draw definitive conclusions. Nevertheless, preliminary findings appear promising and justify continued clinical research. Much of the current rationale for lithium use in BPD is extrapolated from its established efficacy in related psychiatric conditions, such as bipolar disorder, which share overlapping psychopathological features. Despite these limitations, there has been little progress in developing new pharmacological treatments for BPD over the past several decades. Consequently, lithium remains an attractive candidate for further study as a potential therapeutic option in the management of BPD. Continued investigation into its clinical effectiveness could provide not only improved patient outcomes but also a more accessible and cost-effective treatment approach for this challenging mental health condition. Identifying lithium as an effective treatment option for BPD that may both be clinically effective and while it may also provide a more affordable cost-effective approach for this mental health condition is both medically worthy and justified. To determine lithium’s clinical efficacy for BPD demonstrating the extent of the scope of its benefits is recognized to be worthy of further clinical evaluation.
